# Supplementary material for: Screening of Small-Molecule Libraries Using SARS-CoV-2-Derived Sequences Identifies Novel Furin Inhibitors
Source: Int J Mol Sci. 2024 May 7;25(10):5079. doi: 10.3390/ijms25105079 (PMC11121672; doi:10.3390/ijms25105079)
Supplement: Supplementary file 1 [file ijms-25-05079-s001.zip › ijms-2957185-supplementary figures.pdf]

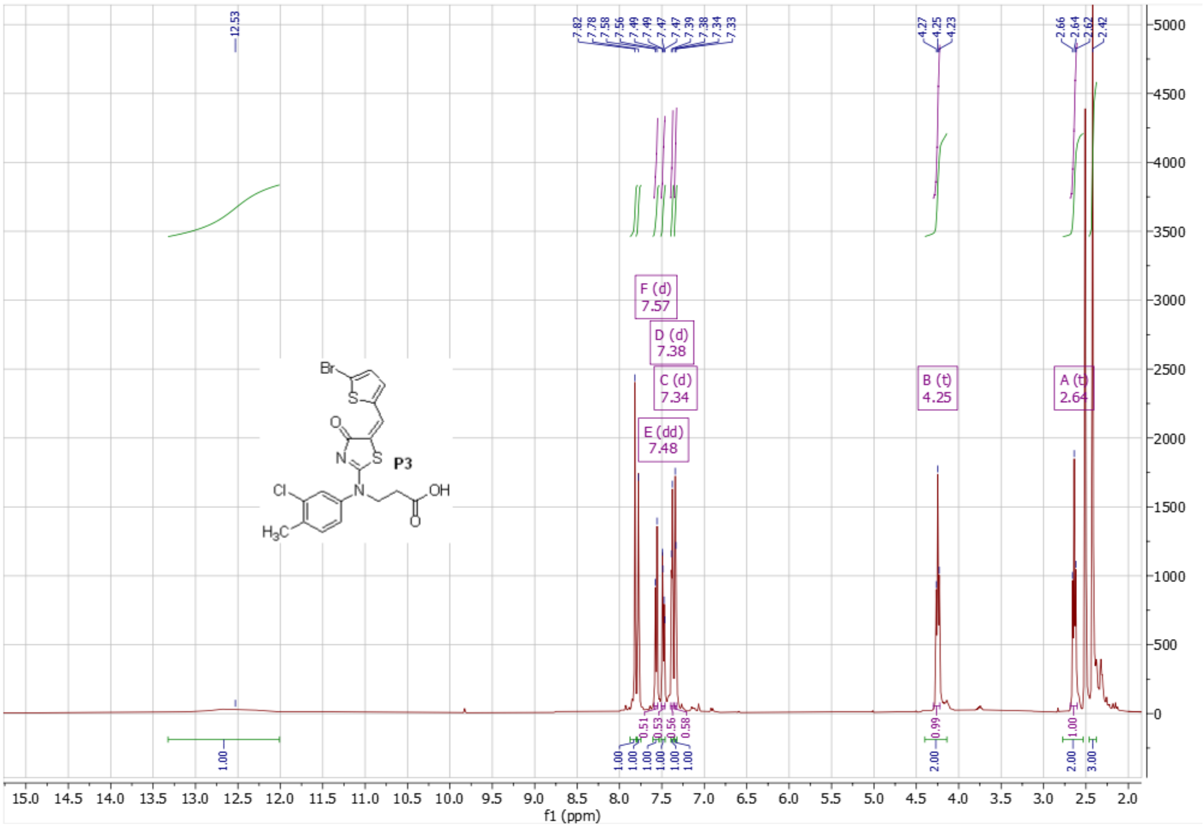

Figure S1 ^{1}H NMR spectrum of compound **P3**

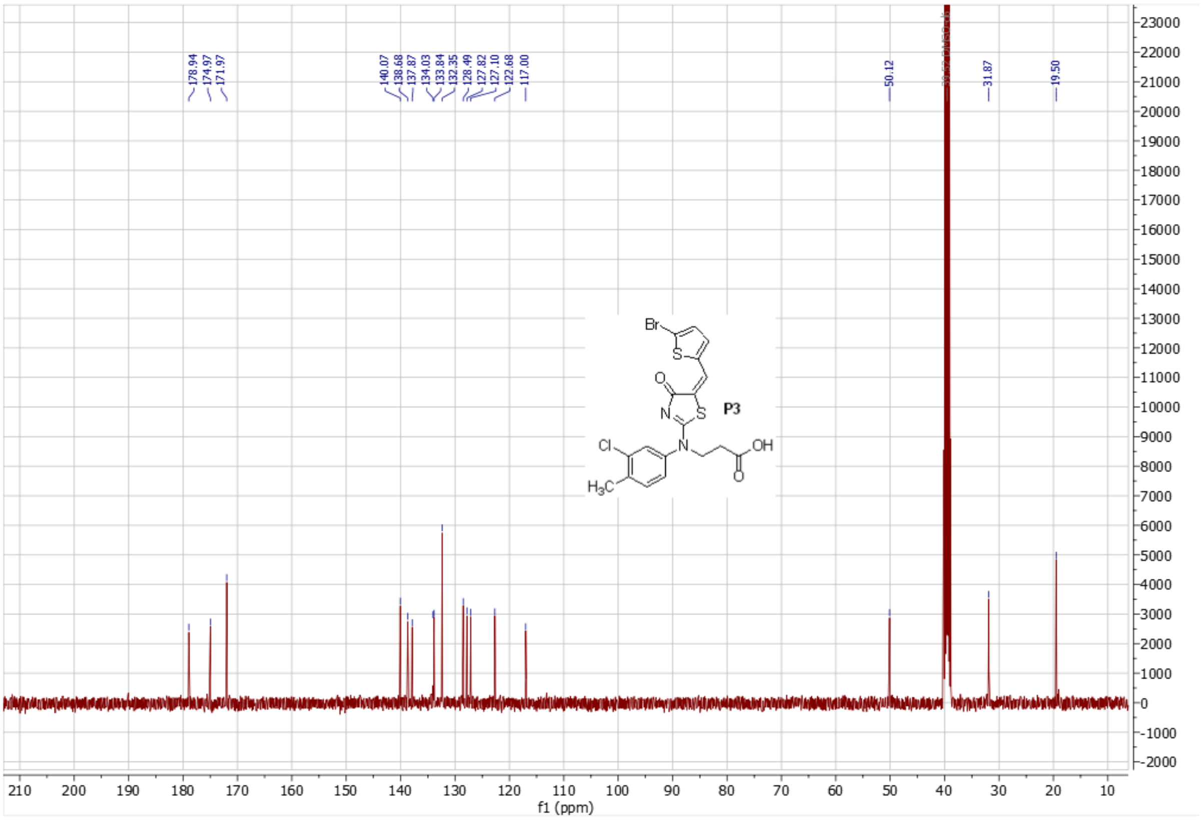

Figure S2 ^{13}C NMR spectrum of compound **P3**

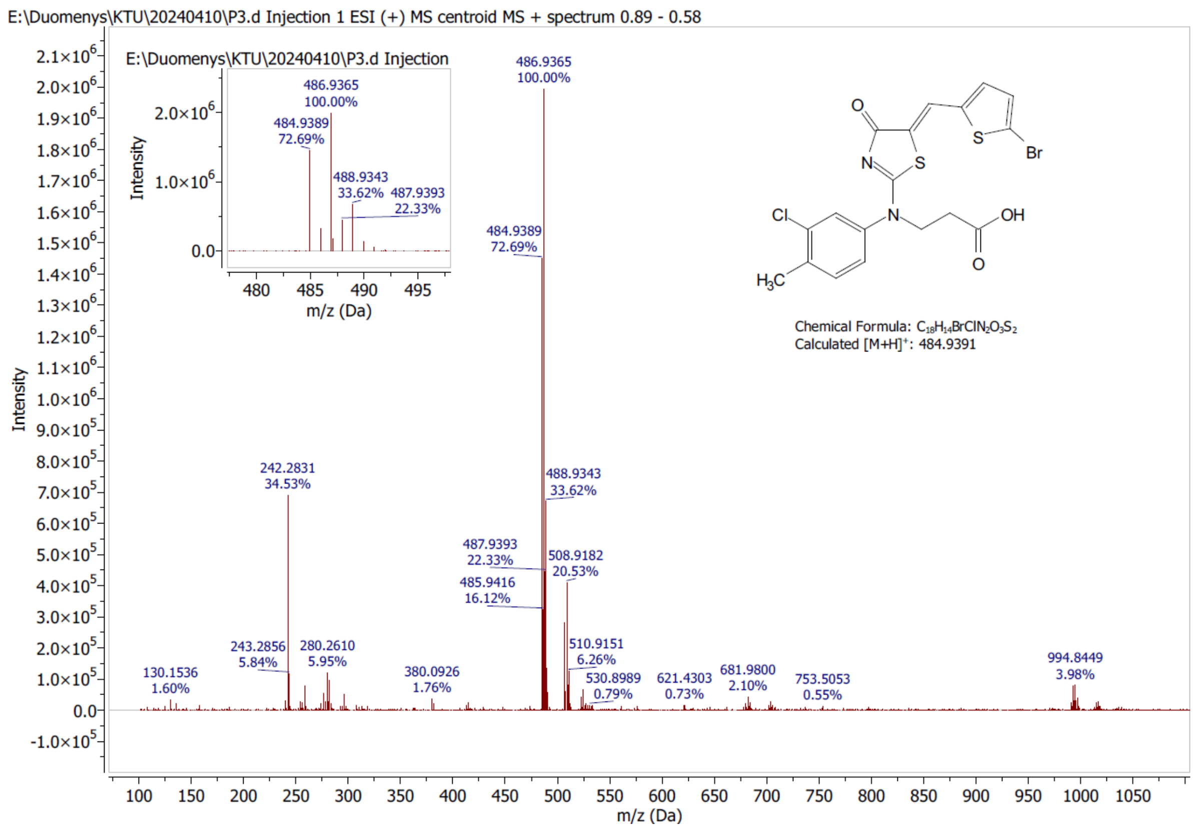

Figure S3 Mass spectrum of compound **P3**

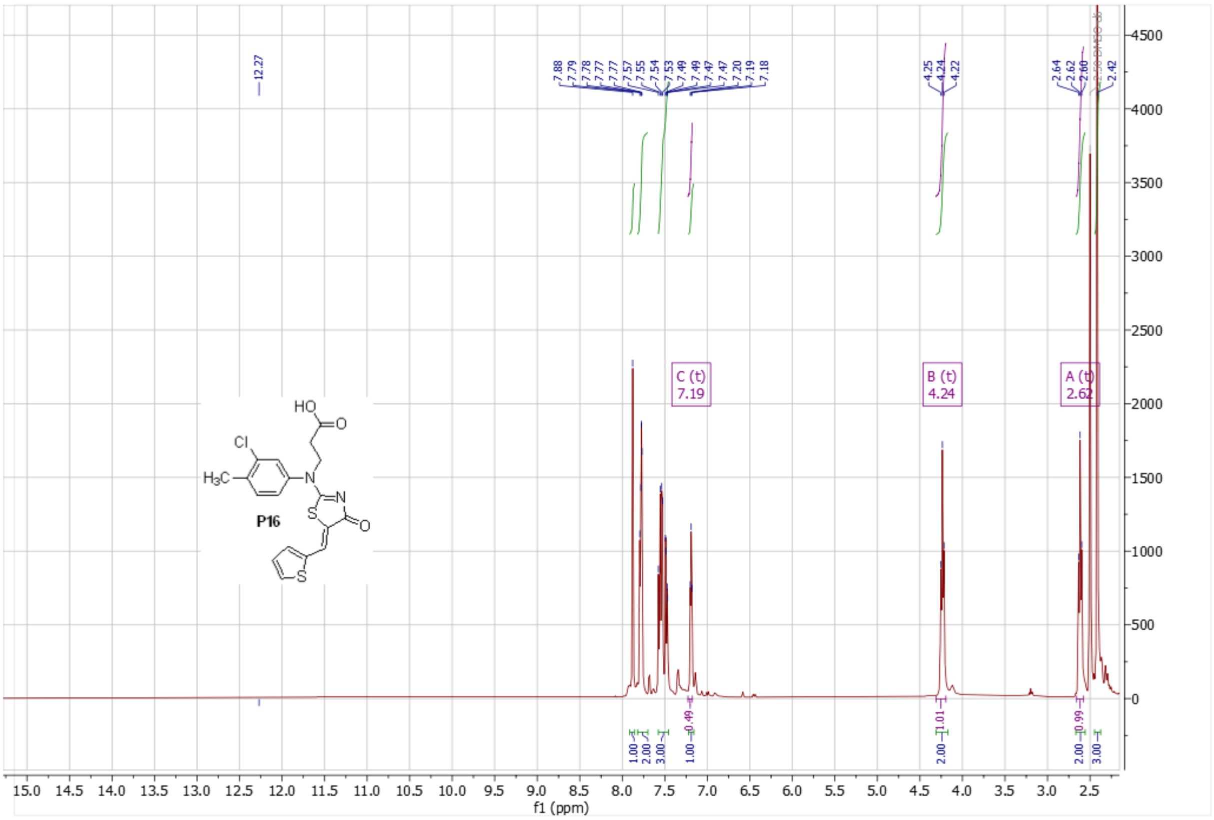

Figure S4 ^{1}H NMR spectrum of compound **P16**

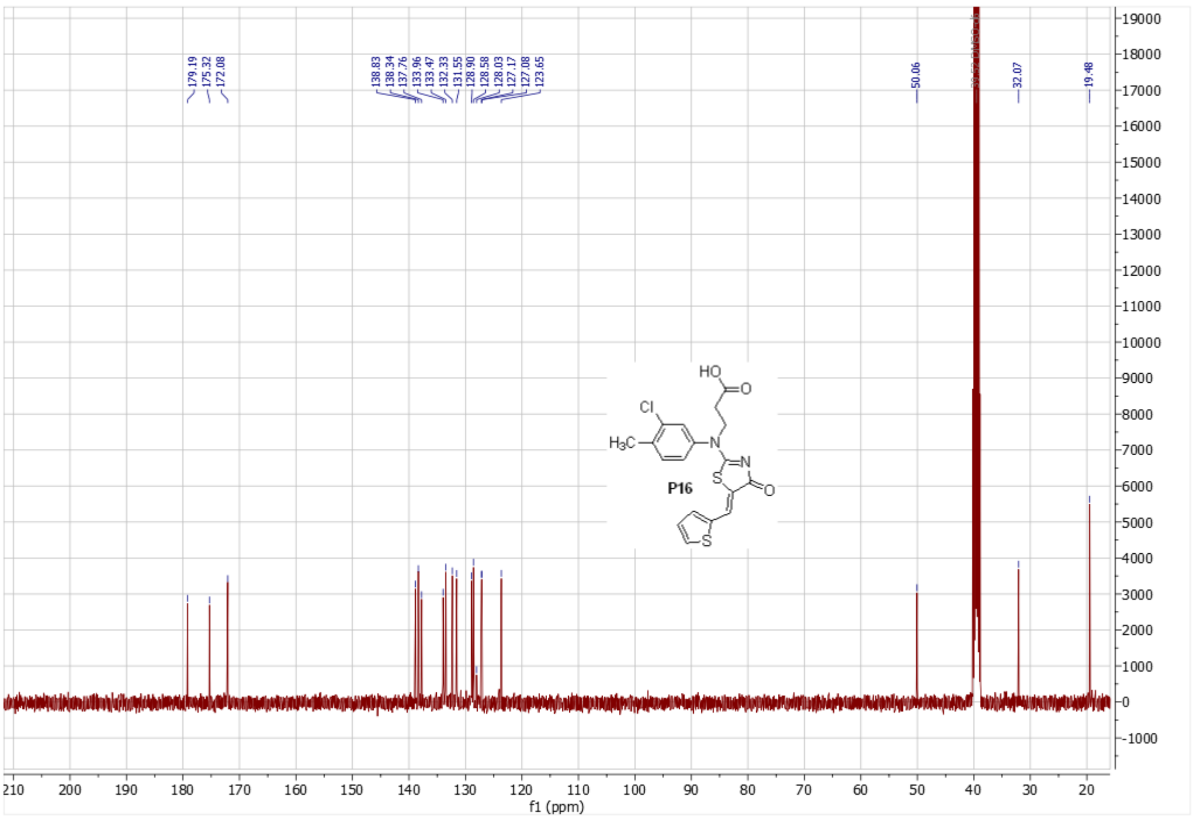

Figure S5 ^{13}C NMR spectrum of compound **P16**

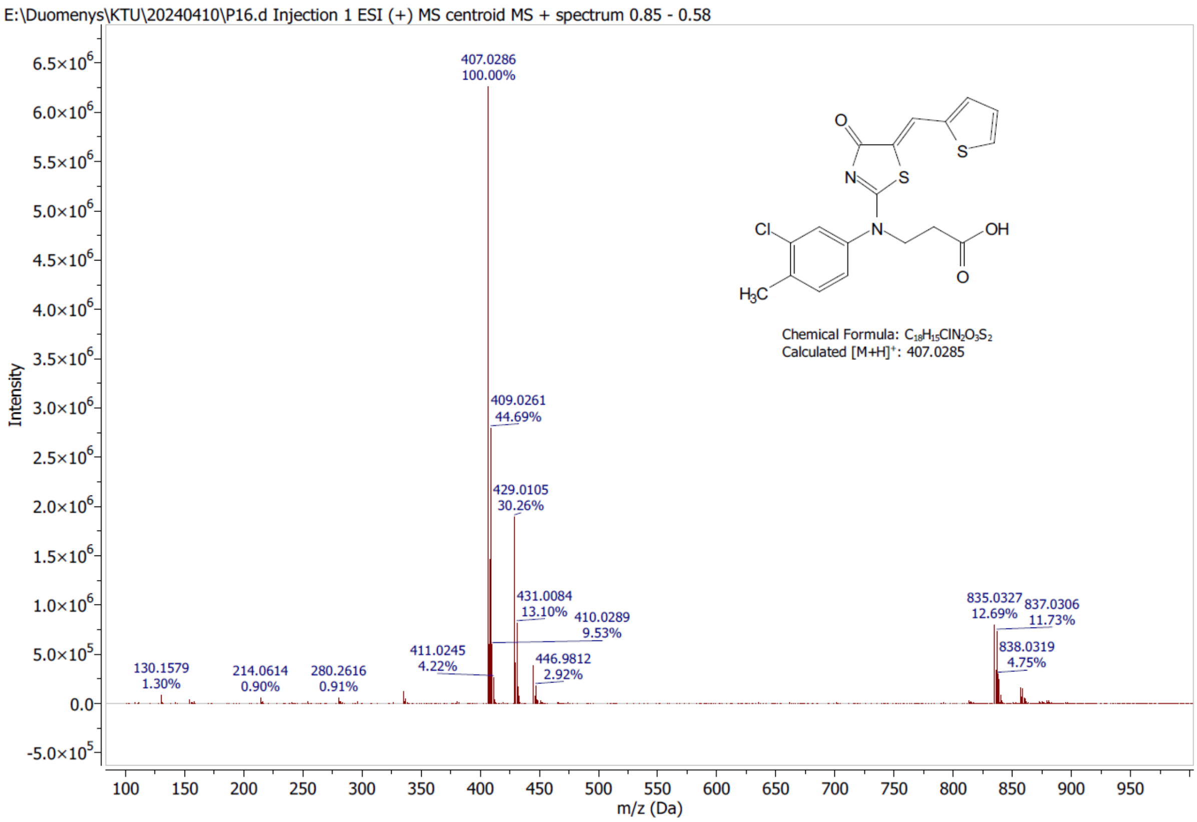

Figure S6 Mass spectrum of compound **P16**

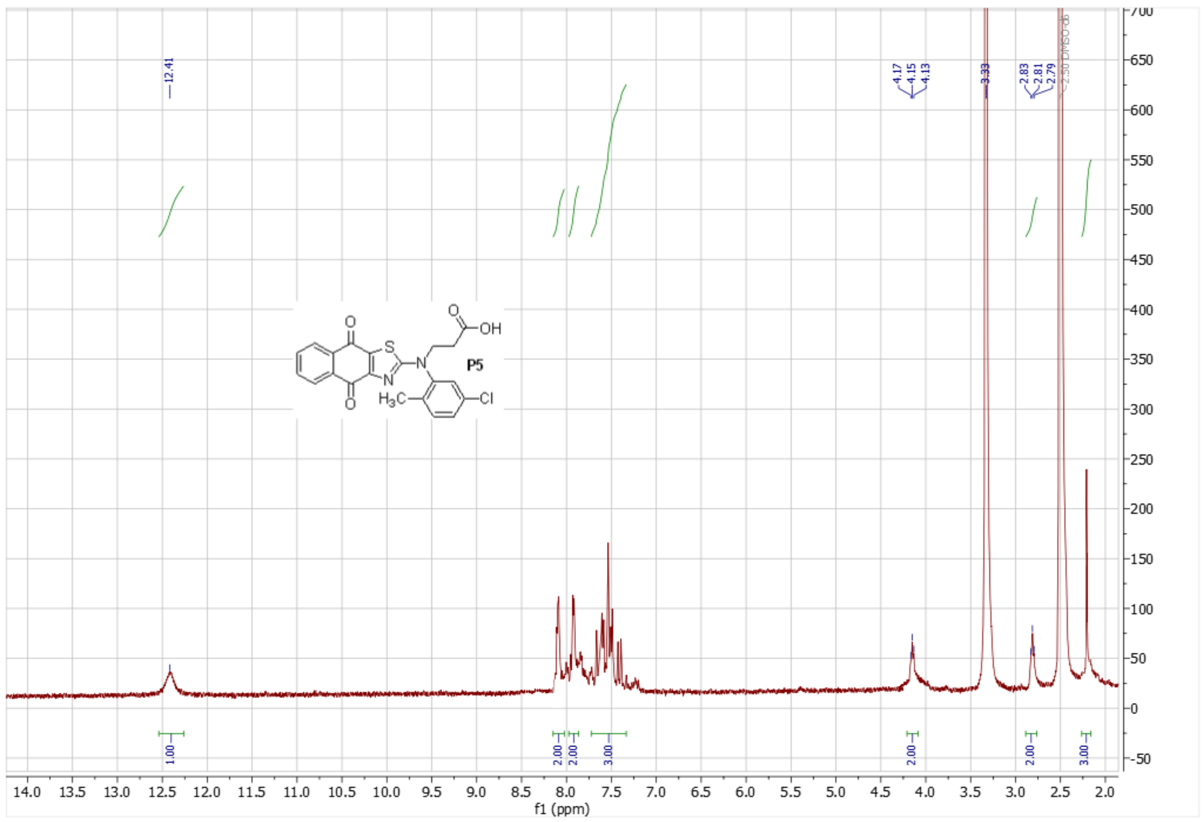

Figure S7 ^{1}H NMR spectrum of compound **P5**

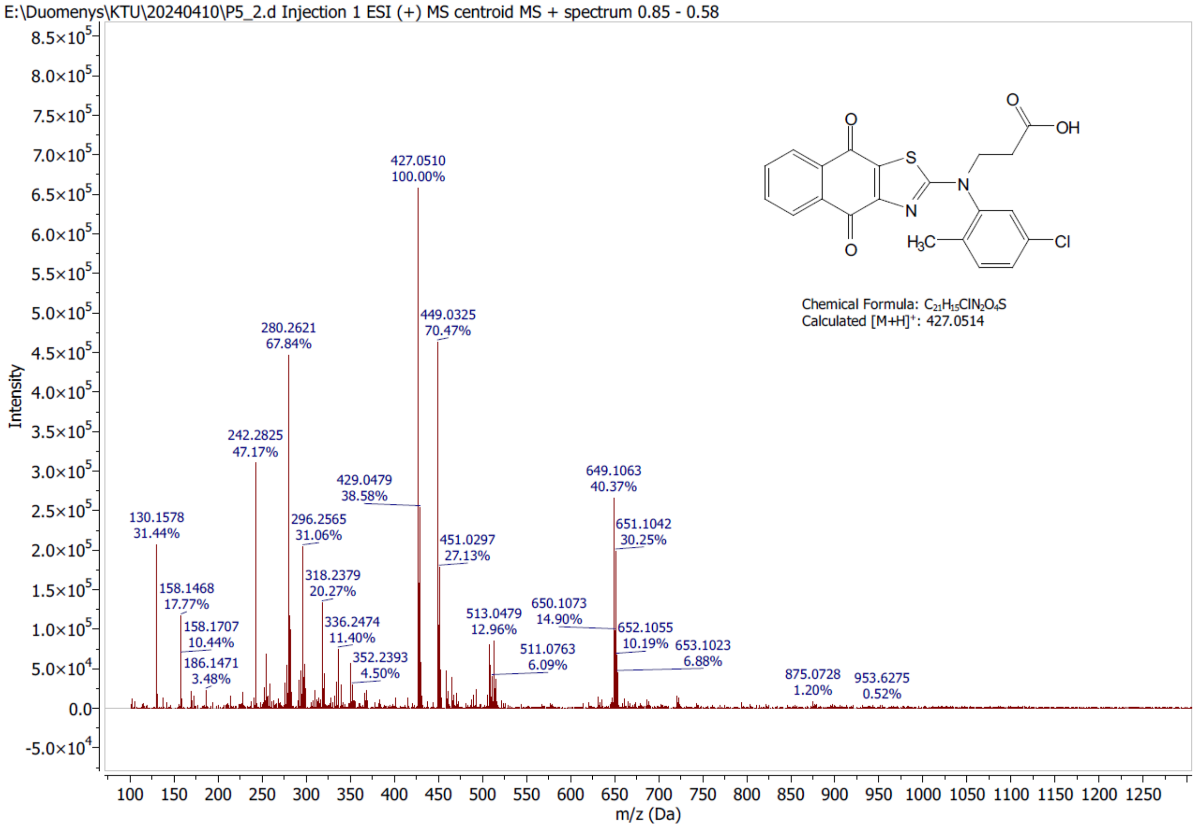

Figure S8 Mass spectrum of compound **P5**

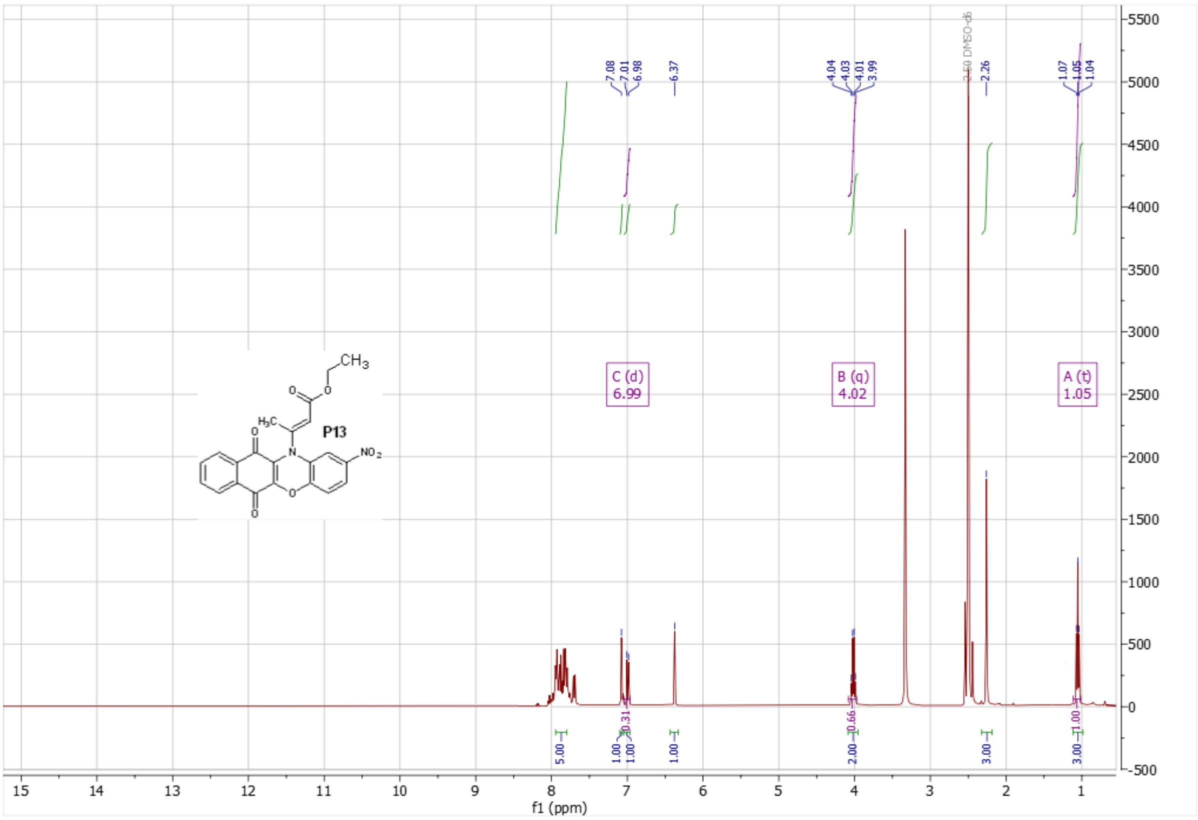

Figure S9 ^{1}H NMR spectrum of compound **P13**

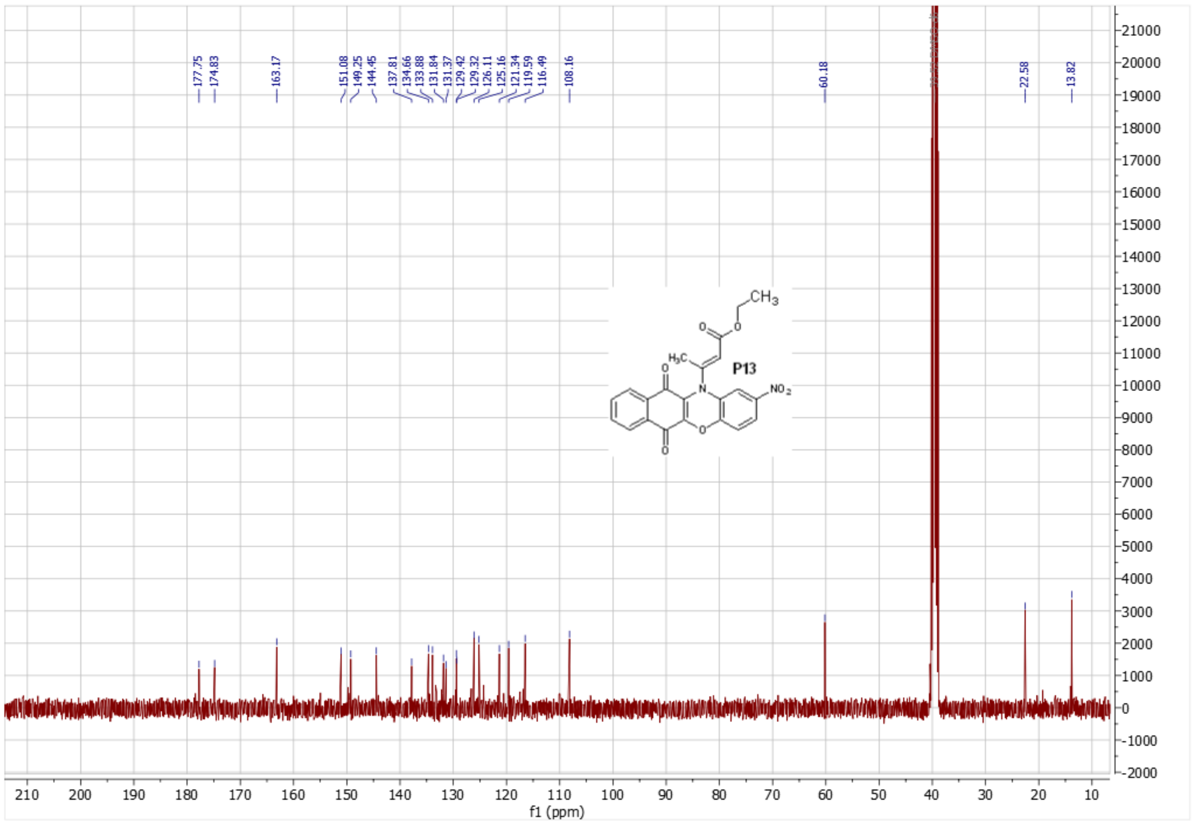

Figure S10 ^{13}C NMR spectrum of compound **P13**

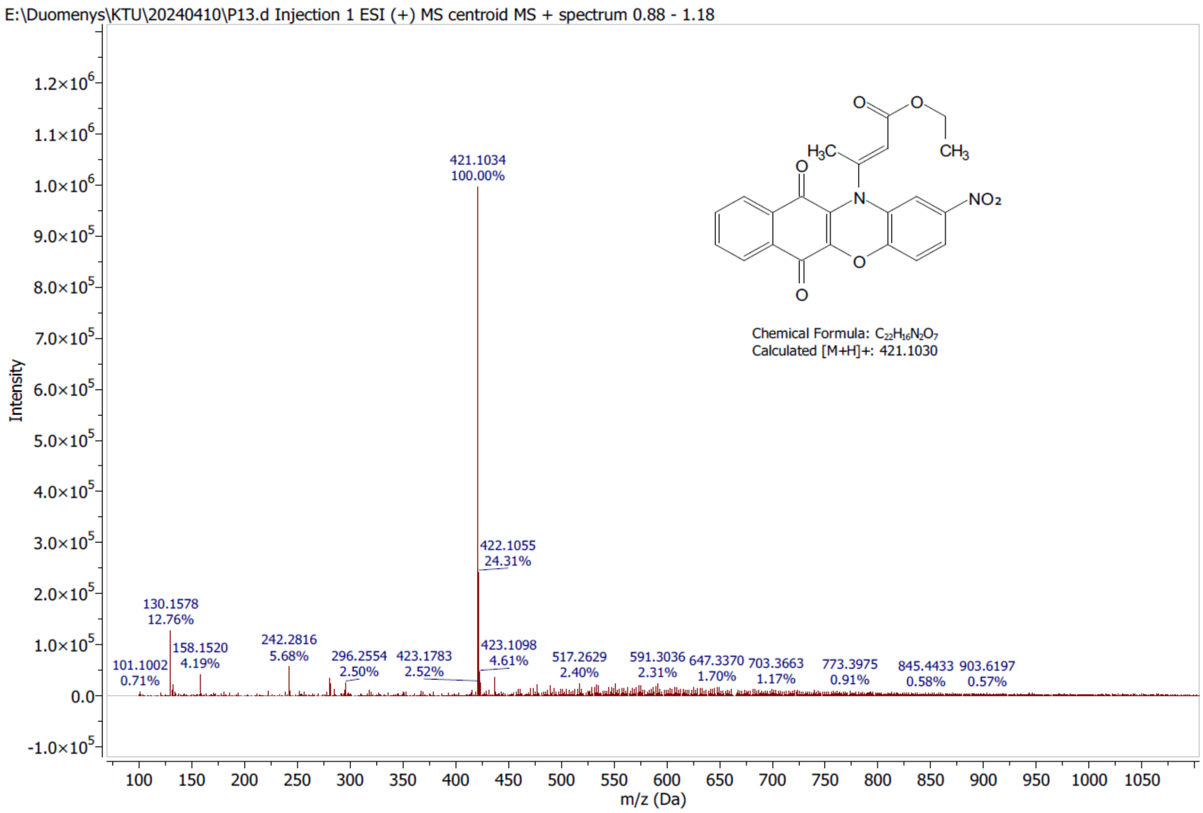

Figure S11 Mass spectrum of compound **P13**
