# Supplementary material for: Screening of Small-Molecule Libraries Using SARS-CoV-2-Derived Sequences Identifies Novel Furin Inhibitors
Source: Int J Mol Sci. 2024 May 7;25(10):5079. doi: 10.3390/ijms25105079 (PMC11121672; doi:10.3390/ijms25105079)
Supplement: Supplementary file 1 [file ijms-25-05079-s001.zip › Table S3.pdf]

Table S3. Plastic items used in the current work and registered in our Green Book (GB)

Object	Quantity	Unit weight [g]	TOT weight [g]
Flask (75cm^2)	7	60,00	420,00
Plate (24 well)	5	65,40	327,00
Plate (48 well)	5	56,00	280,00
Plate (96 well)	40	64,60	2584,00
Sterile pipette (10mL)	50	14,00	700,00
Sterile pipette (25mL)	25	15,60	390,00
Optical clear adhesive sheets	30	2,00	60,00
Sterile pipette (5mL)	40	7,90	316,00
Tips (10 μL)	6000	0,12	720,00
Tips (1000 μL)	500	0,76	380,00
Tips (200 μL)	6000	0,27	1620,00
Tube (0,5 mL)	500	0,48	240,00
Tube (1,5 mL)	700	1,00	700,00
Tube (15 mL)	10	6,40	64,00
Tube (50 mL)	50	12,70	635,00
Glove pairs	200	6,20	1240,00
Other			1000,0
		**TOT**	**11676,00**
